# Bone marrow-derived stem/stromal cells (BMSC) 3D microtissues cultured in BMP-2 supplemented osteogenic induction medium are prone to adipogenesis

**DOI:** 10.1007/s00441-018-2894-y

**Published:** 2018-08-22

**Authors:** K. Futrega, E. Mosaad, K. Chambers, W. B. Lott, J. Clements, M. R. Doran

**Affiliations:** 1 Stem Cell Therapies Laboratory, Queensland University of Technology (QUT), Institute of Health and Biomedical Innovation (IHBI), Translational Research Institute (TRI), Brisbane, Australia; 2 Science and Engineering Faculty (SEF), Translational Research Institute (TRI), Brisbane, Australia; 3 Australian Prostate Cancer Research Centre – Queensland (APCRC-Q), Institute of Health and Biomedical Innovation (IHBI) & School of Biomedical Sciences, Queensland University of Technology (QUT), Translational Research Institute (TRI), Brisbane, Australia; 4 Biochemistry Division, Chemistry Department, Faculty of Science, Damietta University, Damietta, Egypt; 5 Quadram Institute Bioscience, Norwich Research Park, Norwich, UK; 6 Mater Research Institute – University of Queensland (UQ), Translational Research Institute (TRI), Brisbane, Australia; 7 Australian National Centre for the Public Awareness of Science, Australian National University (ANU), Canberra, Australia

**Keywords:** Cell dimensions, Microtissue, Cell spheroid, Bone marrow mesenchymal stromal cells, Tissue regeneration, BMP-2, Osteogenic induction

## Abstract

Bone marrow-derived mesenchymal stem/stromal cells (BMSC) may facilitate bone repair through secretion of factors that stimulate endogenous repair processes or through direct contribution to new bone through differentiation into osteoblast-like cells. BMSC microtissue culture and differentiation has been widely explored recently, with high-throughput platforms making large-scale manufacture of microtissues increasingly feasible. Bone-like BMSC microtissues could offer an elegant method to enhance bone repair, especially in small-volume non-union defects, where small diameter microtissues could be delivered orthoscopically. Using a high-throughput microwell platform, our data demonstrate that (1) BMSC in 3D microtissue culture result in tissue compaction, rather than growth, (2) not all mineralised bone-like matrix is incorporated in the bulk microtissue mass and (3) a significant amount of lipid vacuole formation is observed in BMSC microtissues exposed to BMP-2. These factors should be considered when optimising BMSC osteogenesis in microtissues or developing BMSC microtissue-based therapeutic delivery processes.

**Electronic supplementary material:**

The online version of this article (10.1007/s00441-018-2894-y) contains supplementary material, which is available to authorized users.

## Introduction

Mesenchymal stem/stromal cells (MSC), sometimes referred to as mesenchymal stem cells or mesenchymal stromal cells, are a rare cell population found in the connective tissue of most adult organs, where they appear to play a critical role in tissue repair and regeneration (Bianco 2014). MSC have been most commonly isolated and characterised from bone marrow aspirates (Doran et al. 2010; Bara et al. 2014; Futrega et al. 2017), placenta (Fukuchi et al. 2004; Timmins et al. 2012) and adipose tissue (Zuk et al. 2001). The defining characteristics of MSC include plastic-adherence, expression of specific cell surface markers and the ability to differentiate into the mesodermal cell types, including those of osteogenic, chondrogenic and adipogenic lineages (Dominici et al. 2006). The MSC multipotent differentiation capacity and paracrine secretions suggest that these cells may contribute to tissue repair through (1) differentiation into cells that can directly contribute to new tissue, (2) secretion of factors that may up-regulate endogenous repair processes or (3) secretion of factors that dampen inflammatory processes (Bianco et al. 2008).

Bone marrow-derived MSC (BMSC) are being studied in a range of bone repair applications (Schneider et al. 2010; Seong et al. 2010; Zhang et al. 2010; Gamie et al. 2012). It is thought that BMSC likely promote bone tissue regeneration by differentiating into osteoblasts and by promoting neo-vascularisation, thereby facilitating the growth of new tissue (Schneider et al. 2010; Zhang et al. 2010). Common delivery strategies inject BMSC as single cell suspensions, while others anchor BMSC on scaffolds or in hydrogels for implantation into the bone defect site (Seong et al. 2010; Gamie et al. 2012). BMSC differentiation state and mode of delivery influence the cost and potential efficacy of treatment. The number of potential therapeutic permutations and combinations is vast and as yet, there is no recognised gold standard. Studies that manipulate BMSC as multicellular spheroids or microtissues are increasingly common (Markway et al. 2010; Baraniak and McDevitt 2012; Kabiri et al. 2012; Futrega et al. 2015, 2017), as 3D BMSC microtissue culture exhibit increased maintenance of stem-like qualities and improved differentiation capacity (Mendez-Ferrer et al. 2010; Pinho et al. 2013). Controlling the specific size of microtissues enables optimisation of biological potency while generating a spheroidal cell product with a suitable diameter for injection via a minimally invasive needle or orthoscopic tool (Futrega et al. 2016). Microtissue platforms enable efficient and rapid manufacture of thousands of multicellular microtissues of specifically controlled size (Markway et al. 2010; Futrega et al. 2015, 2017). Relative to single cell injections, microtissues may also reduce loss of cells from the site of delivery and deliver more mature and matrix-rich tissues. The high-throughput capacity of microwell platforms (Markway et al. 2010; Futrega et al. 2015, 2017) makes microtissue-based therapeutic approaches promising and increasingly feasible for clinical applications.

Bone morphogenetic proteins (BMPs) are among the most studied growth factors used to enhance BMSC osteogenesis and bone formation, both in vitro and in vivo (Carlisle and Fischgrund 2005). In 2002, BMP-2 gained humanitarian FDA approval for use in spinal fusion (FDA 2002) and BMP-2 is now commonly used in a range of off label bone repair applications (Hamilton et al. 2008; Ong et al. 2010). The clinical history of BMP-2 and its availability as a clinical grade product, make it an attractive growth factor to consider for attempting to enhance the quality of bone-like microtissues manufactured from BMSC in vitro.

While the influence of BMP-2 on BMSC differentiation has been studied extensively in 2D monolayers (Sun et al. 2015), its effect on 3D microtissue cultures has been less well explored. As undifferentiated BMSC organised into 3D microtissues behave differently than those cultured in 2D monolayers (Wang et al. 2009; Frith et al. 2010; Kabiri et al. 2012), we chose to characterise BMSC osteogenic induction in 3D microtissue cultures. We manufactured hundreds of BMSC microtissues and characterised their differentiation outcome in standard osteogenic medium, osteogenic medium supplemented with BMP-2, adipogenic induction medium, and control medium supplemented with fetal bovine serum (FBS). Using microscopy and a calcium quantification assay, BMSC differentiation was characterised in 2D monolayers and in 3D microtissues by assessing the presence of bone-like mineralised matrix and lipid vacuole accumulation.

## Materials and methods

### BMSC isolation, culture and characterisation

Human bone marrow aspirates were collected at the Mater Hospital (Brisbane, Australia) from fully informed and consenting healthy volunteer donors. In accordance with the Australian National Health and Medical Research Council’s Statement on Ethical Conduct in Research Involving Humans, ethical approval was granted through the Mater Health Services Human Research Ethics Committee and Queensland University of Technology Ethics Committee (number: 1000000938). BMSC donor information was as follows: donor 1 was a 55-year-old male, donor 2 was a 23-year-old male and donor 3 was a 21-year-old female. Aspirates were collected from the iliac crest of volunteer donors. Mononucleated cell isolation was achieved by density gradient centrifugation, using Ficoll-Paque Plus (GE Healthcare), as described previously (Futrega et al. 2017). Bone marrow aspirates were diluted to 35 mL with PBS containing 2 mM EDTA (both from ThermoFisher) and carefully overlayed on top of 15 mL of Ficoll-Paque plus. Following centrifugation for 30 min at 400×*g*, mononuclear cells were collected from the interface, washed with PBS and pelleted at 300×*g* for 5 min. Cell pellets were resuspended in low glucose Dulbecco’s modified Eagle’s medium (DMEM-LG; ThermoFisher) supplemented with 10% fetal bovine serum (FBS; ThermoFisher) and 1% penicillin/streptomycin (PenStrep; ThermoFisher), distributed into five T175 flasks and cultured overnight in a humidified incubator containing 5% CO_2_ with 20% O_2_ atmosphere at 37 °C. Tissue culture plastic-adherent cells were enriched by removing the medium containing non-adherent cells and fresh culture medium was added to each flask. Subsequent BMSC expansion was performed in a 2% O_2_ and 5% CO_2_ atmosphere at 37 °C. Cells were passaged when the monolayer reached approximately 80% confluence using 0.25% Trypsin/EDTA (ThermoFisher). All experiments were performed using BMSC between passage 2 and 5.

The isolated cells were characterised by flow cytometry for the expression of BMSC surface antigens: CD44, CD90, CD105, CD73, CD146, CD45, CD34 and HLA-DR, as we previously described (Futrega et al. 2016). Briefly, cells were trypsinized and stained with fluorescent-conjugated antibodies or isotype controls as per the manufacturer’s instructions (Miltenyi Biotec). Stained cells were washed and resuspended in MACs buffer (Miltenyi Biotec) and then flow cytometry was performed on a Fortessa flow cytometer (BD Biosciences). Data were analysed using FlowJo software (TreeStar, USA).

### Microwell plate fabrication

The fabrication of polydimethylsiloxane (PDMS, Slygard) microwell arrays was performed as described previously (Chambers et al. 2014; Futrega et al. 2015). Briefly, liquid PDMS (1:10 curing agent to polymer ratio) was permitted to cure over a patterned polystyrene mould having the negative of the desired microwell pattern for 1 h at 80 °C. The sheet of cured PDMS with the microwell array pattern cast into it was peeled away from the mould. This moulding technique produced PDMS microwells with dimensions of 800 × 800 μm in length and width and 400 μm in depth. PDMS discs of ~ 1 cm^2^ were punched from the PDMS sheets using a wad punch. Individual 1-cm^2^ microwell inserts were then anchored into 48-well culture plates (Nunc) using a small dab of Selly’s Aquarium Safe silicone glue. Plates with microwell inserts were submerged in 70% ethanol for 1 h for sterilisation, followed by 3 rinses with sterile deionised water, with a final soak for 1 h. For storage, the plates were dried overnight at 60 °C and stored at room temperature in a sterile container until use. To prevent cell adhesion to the PDMS during culture, the PDMS microwell inserts were rinsed with 0.5 mL of sterile 5% Pluronic-F127 (Sigma-Aldrich) solution for 5 min and then rinsed 3 times with PBS before cell seeding. To expel any visible bubbles from microwells during the sterilisation and rinsing procedure, the plates were centrifuged at 2000*×g* for 2–5 min.

### 2D and 3D culture establishment

**Fig. 1:**
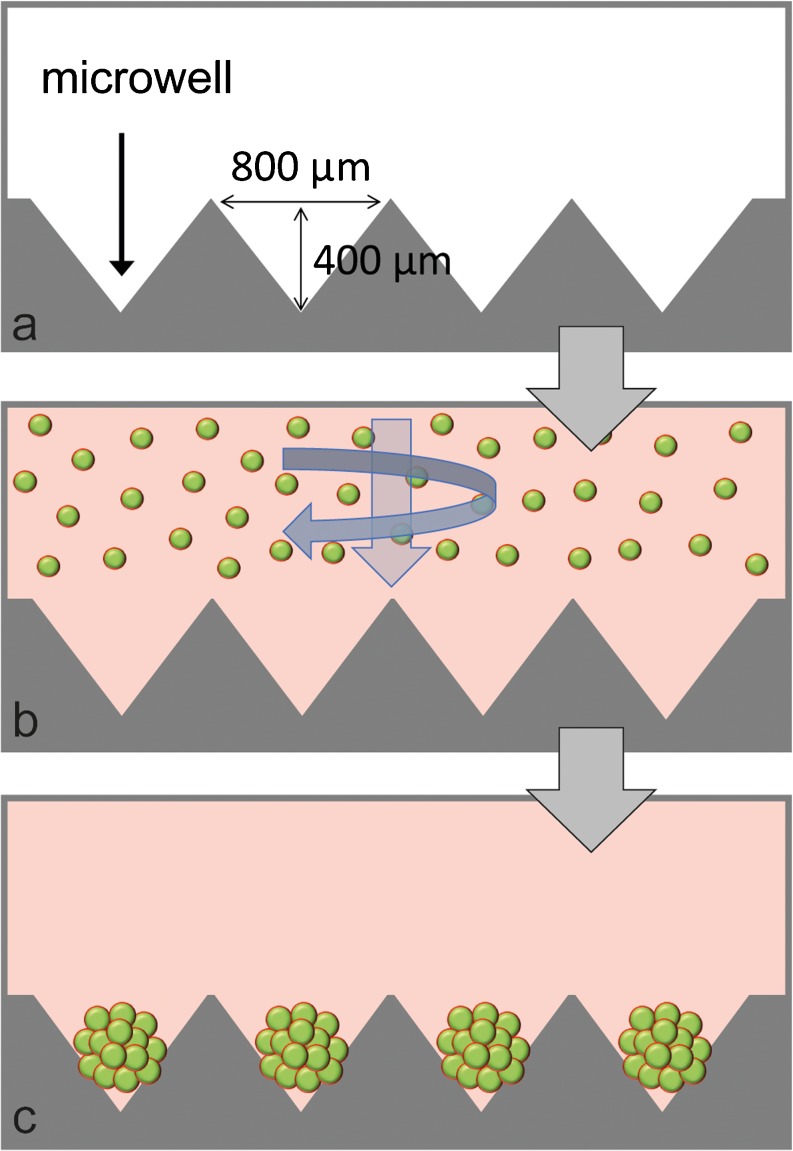
Schematic of microwell platform for microtissue formation. The dimensions of microwells are shown (a). Single cell suspensions were centrifuged into microwells (b), resulting in the formation of uniformly sized microtissues (c)

Single cell suspensions were added to plates with or without microwell inserts to form 3D microtissues or 2D monolayers, respectively. Figure 1 provides a schematic of the microwells and shows the assembly of BMSC into microtissues using the microwell platform. Each well in a 48-well plate inserted with a PDMS patterned-disc contained approximately 150 microwells. Adjusting the total number of cells added in suspension over the PDMS inserts during the seeding process controlled the number of cells per microtissue. Unless specified otherwise, 1.5 × 10^5^ BMSC were seeded in 0.5 mL of media over the microwells, yielding ~ 150 microtissues of approximately 1000 BMSC each. Control monolayers were established by seeding 3 × 10^4^ BMSC into single wells in 48-well plates.

### BMSC monolayer and microtissue differentiation

In this assay, microtissues were manufactured from 1000 BMSC each. This number was selected following optimisation studies using microtissues of 200, 400, 600, 800, 1000 or 2000 BMSC per microtissue (see Supplementary Fig. 1). BMSC monolayer and microtissue differentiation was induced using standard osteogenic or adipogenic medium formulations. Osteogenic medium contained high-glucose DMEM (DMEM-HG), 10% FBS, 100 μM sodium pyruvate, × 1 Gluta-Max, PenStrep, 100 nM dexamethasone, 50 μM L-ascorbic acid 2-phosphate (Sigma-Aldrich) and 10 mM β-glycerol phosphate (Sigma-Aldrich). Where specified, osteogenic medium was supplemented with 10 ng/mL BMP-2 (INFUSE Bone Graft Kit, Medtronic). Adipogenic medium consisted of DMEM-HG, 100 μM sodium pyruvate, × 1 Gluta-Max, PenStrep, 10% FBS, 10 μg/ml insulin, 100 nM dexamethasone, 200 μM indomethacin and 500 μM 3-isobutyl-1-methyl xanthine (last three components where from Sigma-Aldrich). Maintenance medium contained DMEM-HG, 10% FBS, × 1 Gluta-Max, PenStrep and 100 μM sodium pyruvate. For all cultures, medium was exchanged twice per week and cultures were maintained for 21 days in a 20% O_2_ and 5% CO_2_ incubator.

### Characterisation of BMSC microtissue size and cell size with time

To characterise how BMSC behave in microtissue culture in standard maintenance medium (DMEM-HG + 10% FBS), BMSC were seeded into microwells at densities to yield microtissues containing 300 or 600 BMSC each and cultured for 14 days. At 1, 3, 5, 7, 9, 12 and 14 days, images of microtissues were captured using an Olympus DP26 digital microscope camera and the diameters of 50 microtissues per well were measured using cellSens Software (Olympus). Microtissues were collected for DNA quantification at the same time points.

The diameter of single cells in 3D microtissues and in 2D monolayers was estimated using flow cytometry on day 1 and day 8 of culture. On the day of collection, microtissues and monolayers were dissociated using Trypsin/EDTA at 37 °C for approximately 30 min. Microtissues were pipetted up and down during trypsinization to ensure dissociation into a single cell suspension. Cell digests were passed through a cell strainer (35 μm) to remove large particulate matter. Cells were resuspended in MACs buffer (Miltenyi Biotec) and analysed using a CytoFlex flow cytometer (Beckman Coulter). Relative cell size was estimated using reference latex particles with defined size (CC Size Standard L20, L10 and L5; Beckman Coulter). Data were analysed using FlowJo software.

### DNA and calcium quantification

To measure DNA content in cultures, the Quant-iT PicoGreen dsDNA assay (ThermoFisher) was performed as per the manufacturer’s instructions. Cells were lysed using 0.1% Triton-× 100 (Sigma-Aldrich) in × 1 Tris/EDTA kit buffer. Samples were reacted with the PicoGreen reagent in black 96-well plates and fluorescence read on a plate reader (BMG Omega LABTECH). Calcium content was measured using the o-cresolphthalein complex one (OCPC) colorimetric method as previously described (van den Dolder et al. 2003). Briefly, media was removed from cultures and calcium was extracted by incubating overnight in 0.25 mL of 0.5 N acetic acid, with shaking. Ten microlitres of calcium sample extracts were reacted with 200 μL of o-cresolphthalein sample solution in transparent 96-well plates. A standard curve was generated using serial dilutions of CaCl_2_ (1–200 mg/mL). After incubation at room temperature for 10 min, the plate was read at 575 nm on a Multiskan Go Spectrophotometer (ThermoFisher).

### Microscopic characterisation of microtissues and monolayers

For microscopy analysis, cultures were fixed in 4% paraformaldehyde (PFA; Sigma-Aldrich) for 30 min at room temperature. To characterise bone-like calcium deposits in monolayers, cultures were stained with Alizarin Red S (Sigma-Aldrich). To identify hydroxyapatite deposits, monolayers and microtissues were stained using the OsteoImage kit (Lonza) as per the manufacturer’s instructions. To identify lipid vacuoles, monolayers and microtissues were stained with Oil Red O (Sigma-Aldrich). Where indicated, nuclei were stained with 4′,6-Diamidino-2-Phenylindole (DAPI; ThermoFisher). Monolayer and microtissue bright field microscope images were captured using an Olympus microscope (Model CKX41 equipped with X-Cite 120Q Fluorescence Illuminator). OsteoImage and Oil Red O 3D microtissue images were captured using a confocal microscope (FV1200, Olympus).

### Statistical analysis

Initial BMSC microtissue dimension and cell size characterisation studies were performed using two different BMSC donors. BMSC differentiation studies in monolayer and microtissues were performed using three different BMSC donors (donor information described above). Quantitative data represent the average of 4 biological replicate cultures for each donor, unless otherwise stated. Error bars represent one standard deviation. Statistical significance of data was evaluated using two-way analysis of variance (ANOVA), or *t* tests where specified, in Prism software (version 7.0, GraphPad).

## Results

### BMSC characterisation by flow cytometry

**Fig. 2:**
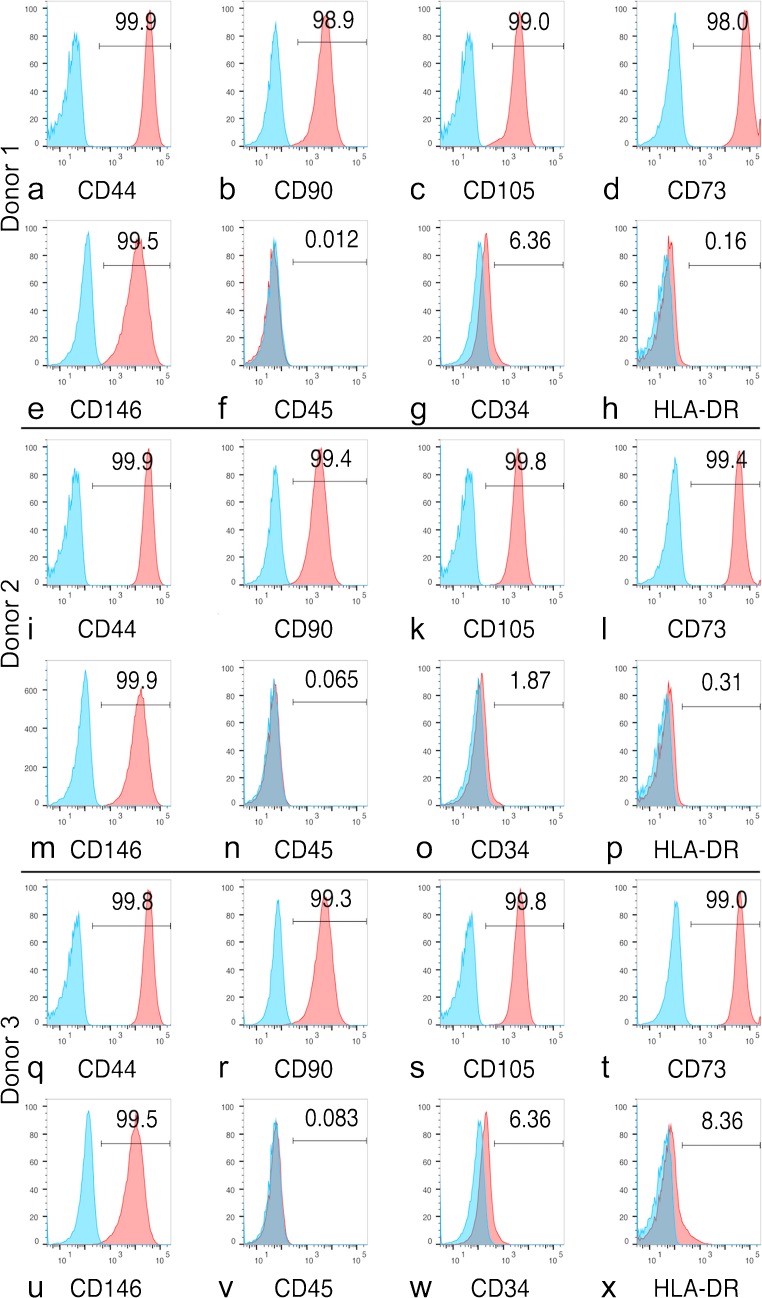
Flow cytometry characterisation of BMSC donor 1 (a–h), donor 2 (i–p) and donor 3 (q–x). Cells were positive for CD44, CD90, CD105, CD73 and CD146 and negative for CD45, CD34 and HLA-DR

Cells stained > 95% positive for BMSC-associated markers (CD44, CD90, CD105, CD73 and CD146) and essentially lacked the expression of haematopoietic markers (CD45 and CD34) and HLA-DR (Fig. 2).

### Characterisation of BMSC culture in 3D microtissues

**Fig. 3:**
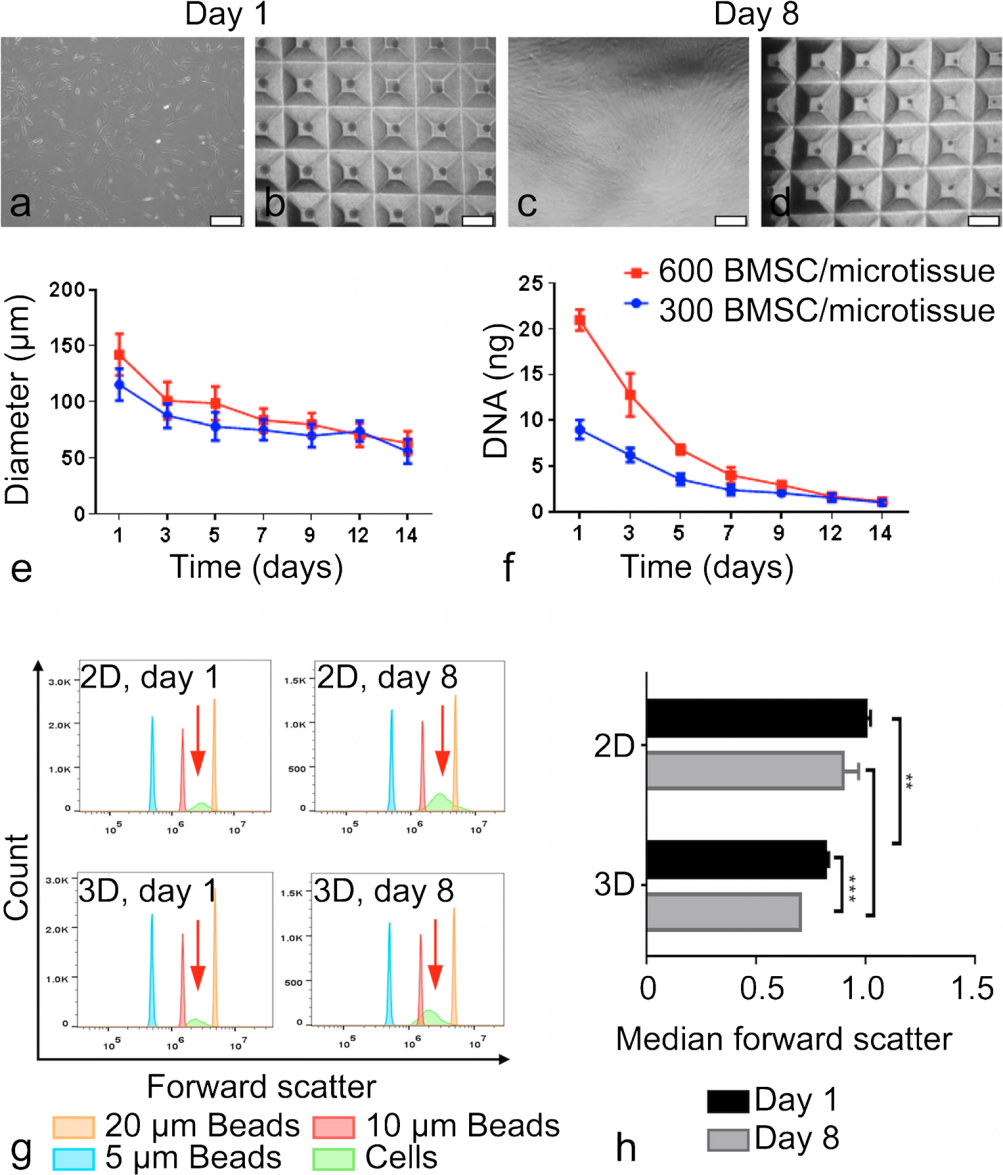
Size characterisation of BMSC in microtissue culture. Bright field images of monolayer (a, c) and microtissue cultures (b, d) on days 1 and 8. Diameter (e) and DNA content (f) of microtissues formed from 300 or 600 BMSC each and cultured in maintenance media. Forward scatter flow cytometry was used to estimate relative cell size, with reference particles having diameters of 5, 10 or 20 μm (g). The bar graph represents the median values of 4 replicate cultures normalised to cells from day 1, 2D monolayer cultures (h). Statistical significance of data was evaluated using two-way analysis of variance (ANOVA, ** *P* < 0.001 and *** *P* < 0.0001). 2D monolayer scale bars = 500 μm and 3D microtissue scale bars = 1 mm

Barniak and McDevitt originally described the compaction of BMSC microtissues (Baraniak and McDevitt 2012), observing that the diameter of microtissues formed from 300 or 600 BMSC each declined over the 7-day culture period and that BMSC proliferation was localised to cells at the periphery of microtissues. Proliferation at the outer edge of microtissues or spheroids, rather than within the core, is commonly reported (Sutherland 1988; Chambers et al. 2014). Here, we report similar observations, in which both microtissue diameter (Fig. 3e) and total DNA content (Fig. 3f) steadily decline when BMSC microtissues were cultured in maintenance media over a 14-day period and proliferation localised at the outer edge of the microtissues (Supplementary Fig. 2). By day 14, BMSC proliferation in microtissues had ceased. To further investigate how 3D microtissue culture impacted the size of individual BMSC, we used Forward Scatter on a flow cytometer and reference beads of defined size to compare the relative cell diameters in 2D monolayer and 3D microtissues after a single day of culture (day 1) and after an additional 1 week of culture (day 8) (Fig. 3g–h). We observed the average cell size decline with time in microtissue cultures to a greater extent than in 2D monolayer cultures. Cell diameter was significantly reduced in microtissue cultures at both time points, consistent with previous reports of reduced cell size in spheroid cultures (Bartosh et al. 2010; Baraniak and McDevitt 2012).

### Osteogenic BMSC differentiation in 2D and 3D cultures

**Fig. 4:**
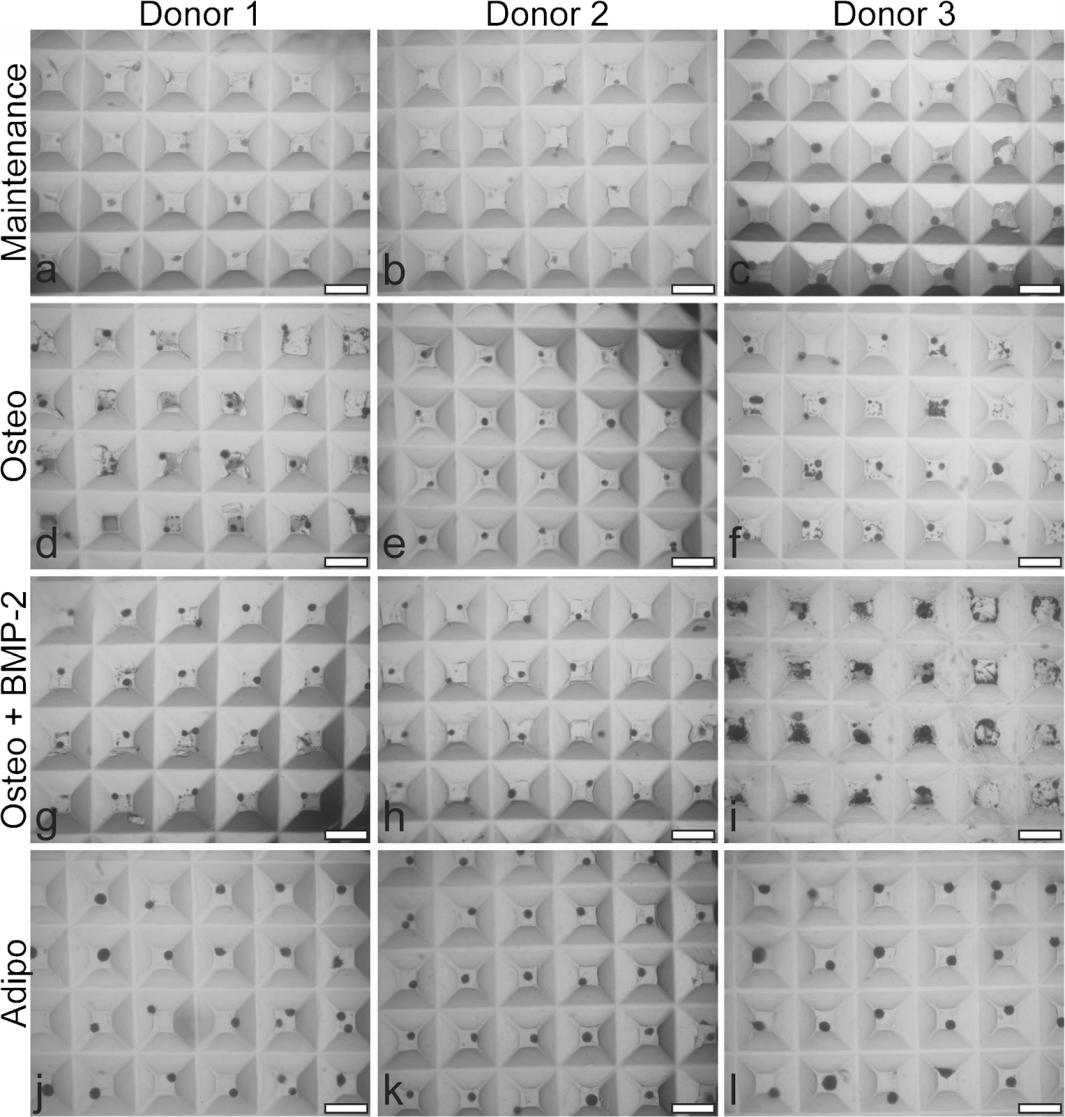
Bright field images of BMSC donor 1 (a, d, g and j), donor 2 (b, e, h and k) and donor 3 (c, f, i and l) microtissues cultured for 21 days in maintenance medium (DMEM + 10% FBS) (a–c), osteogenic medium (Osteo) (d–f), osteogenic medium + BMP-2 (Osteo + BMP-2) (g–i) or adipogenic medium (Adipo) (j–l). Mineral debris can be seen in the microwells beside microtissues cultured in osteogenic or osteogenic medium + BMP-2 medium. Scale bar = 1 mm

Microtissues cultured in maintenance, osteogenic, osteogenic + BMP-2 or adipogenic medium are shown in Fig. 4. Adipogenic microtissues were visually larger than microtissues cultured in other medium formulations. The accumulation of mineralised matrix (dark particles) in osteogenic-induced microtissue cultures, plus or minus BMP-2, was observed both within microtissues and in the space surrounding individual microtissues. By visual inspection, donor 3 showed the earliest and greatest mineralised matrix accumulation (data not shown), followed by donor 1 and then donor 2. This pattern suggested that not all mineralised matrix was retained within the bulk microtissue masses and that mineralisation varied among BMSC donors/cultures.

**Fig. 5:**
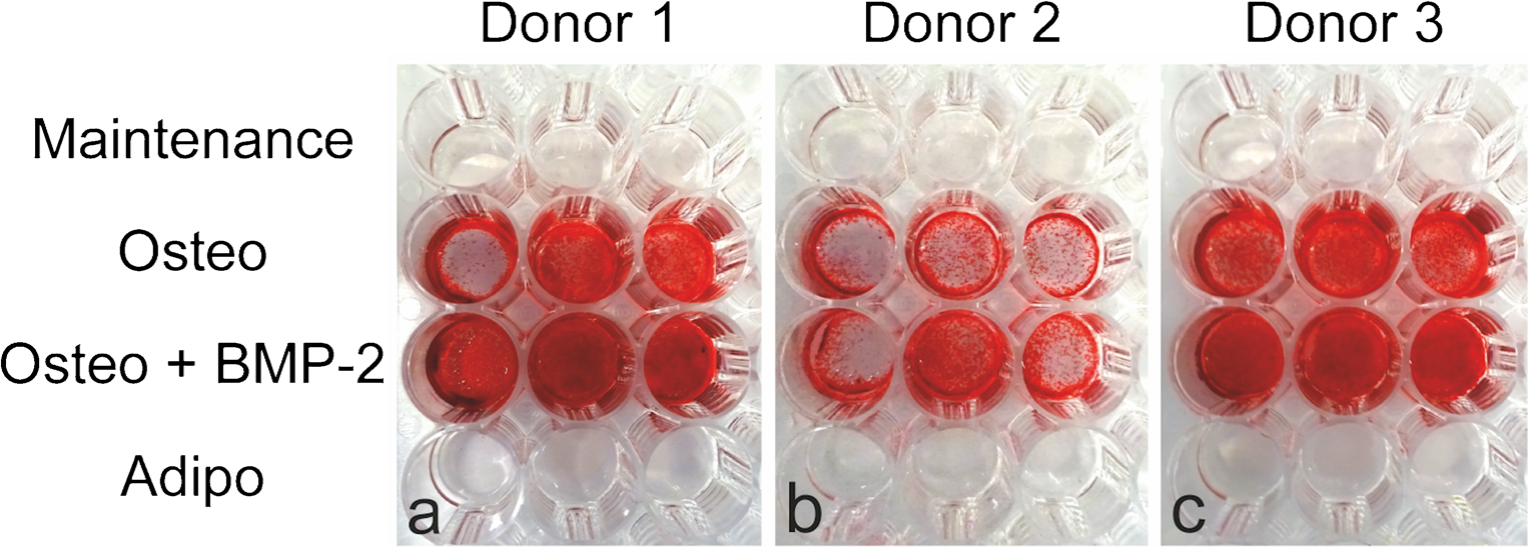
Alizarin Red S staining of monolayer cultures in 48-well plates for BMSC donor 1 (a), donor 2 (b) and donor 3 (c), following 21 days culture in maintenance medium (DMEM + 10% FBS), osteogenic medium (Osteo), osteogenic medium + BMP-2 (Osteo + BMP-2) or adipogenic medium (Adipo)

To detect mineralisation, monolayer cultures were stained with Alizarin Red S and well plates were photographed (Fig. 5). Alizarin Red S staining was positive for monolayer cultures from all BMSC donors cultured in osteogenic induction medium, plus or minus BMP-2. Staining was generally more intense in cultures supplemented with BMP-2; however, staining was not homogenous across all replicate wells and this observation must be cautiously interpreted. No staining was visible on monolayers cultured in either maintenance or adipogenic induction medium.

**Fig. 6:**
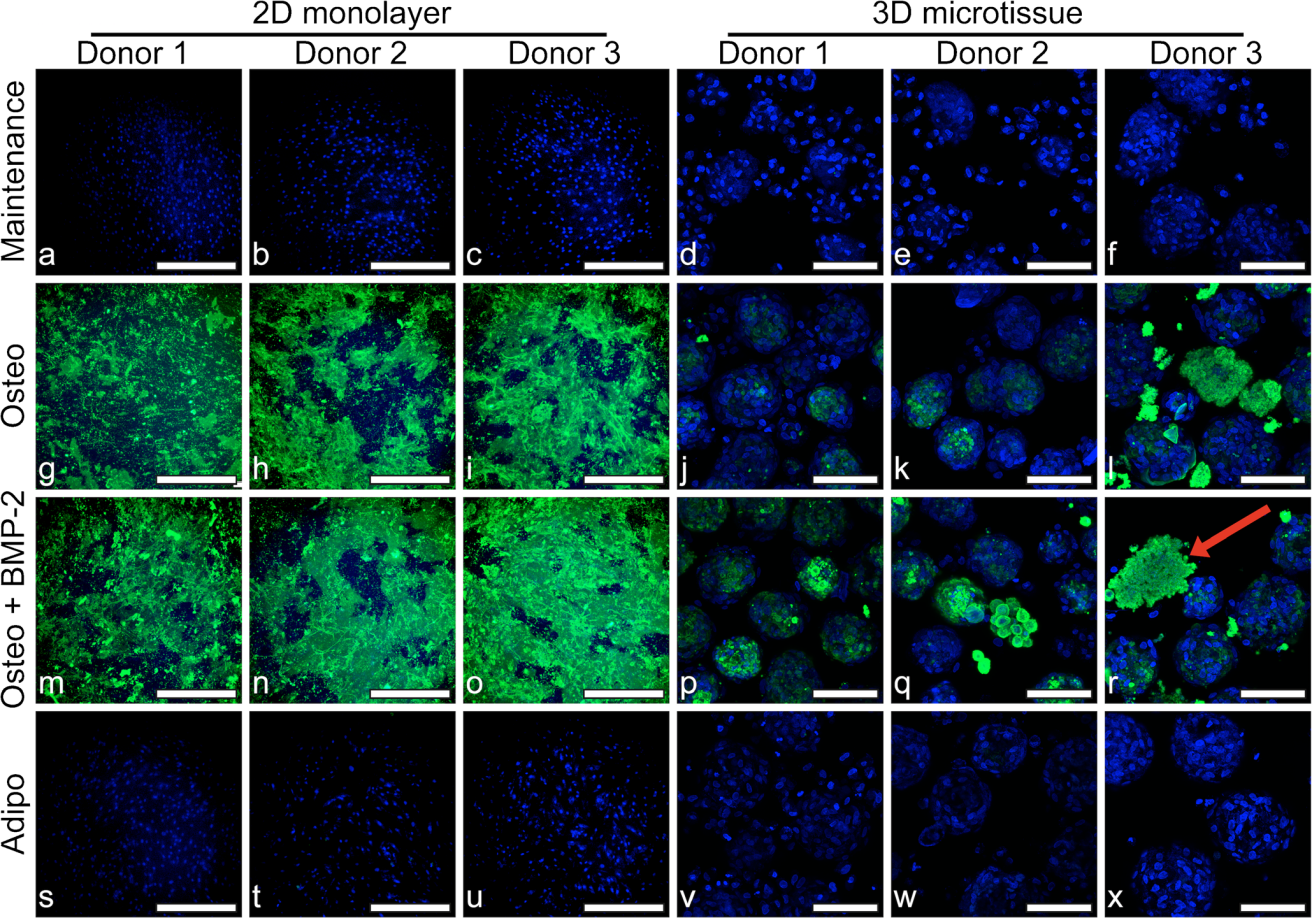
Fluorescence microscopy of 2D cultures for BMSC donor 1 (a, g, m and s), donor 2 (b, h, n and t) and donor 3 (c, i, o and u) and in 3D cultures for BMSC donor 1 (d, j, p and v), donor 2 (e, k, q and w) and donor 3 (f, l, r and x) at day 21. BMSC were cultured in maintenance medium (DMEM + 10% FBS) (a–f), osteogenic medium (Osteo) (g–l), osteogenic medium + BMP-2 (Osteo + BMP-2) (m–r) or adipogenic medium (Adipo) (s–x). Hydroxyapatite deposition was evaluated by staining with OsteoImage (green) and nuclei are stained with DAPI (blue). The red arrow in (r) points to an example of mineralised matrix from a 3D microtissue culture with fragmented nuclear staining. 2D monolayer scale bars = 500 μm and 3D microtissue scale bars = 100 μm

Both 2D monolayer and 3D microtissue cultures were stained with OsteoImage to identify hydroxyapatite deposition (Fig. 6). Similar to Alizarin Red S staining, hydroxyapatite staining was intense in BMSC monolayers cultured in osteogenic media, plus or minus BMP-2 but absent in monolayers cultured in maintenance or adipogenic induction medium. Similar to the microscopy images in Fig. 4, where dark particles could be seen accumulating in and around microtissues in osteogenic medium, hydroxyapatite-rich matrix was observed in confocal images (Fig. 6). DAPI stained nuclei within hydroxyapatite-rich matrix appeared strikingly fragmented in 3D microtissues, suggesting that mineralisation likely results in, or is the result of, cell death. The pattern of hydroxyapatite staining with OsteoImage in 2D monolayers (Fig. 6) was similar to Alizarin Red S staining (Fig. 5) but revealed a more crystalline-like detail. Hydroxyapatite staining was heterogeneous in both monolayer and microtissue cultures, which was likely a result of heterogenous mineral crystal nucleation.

**Fig. 7:**
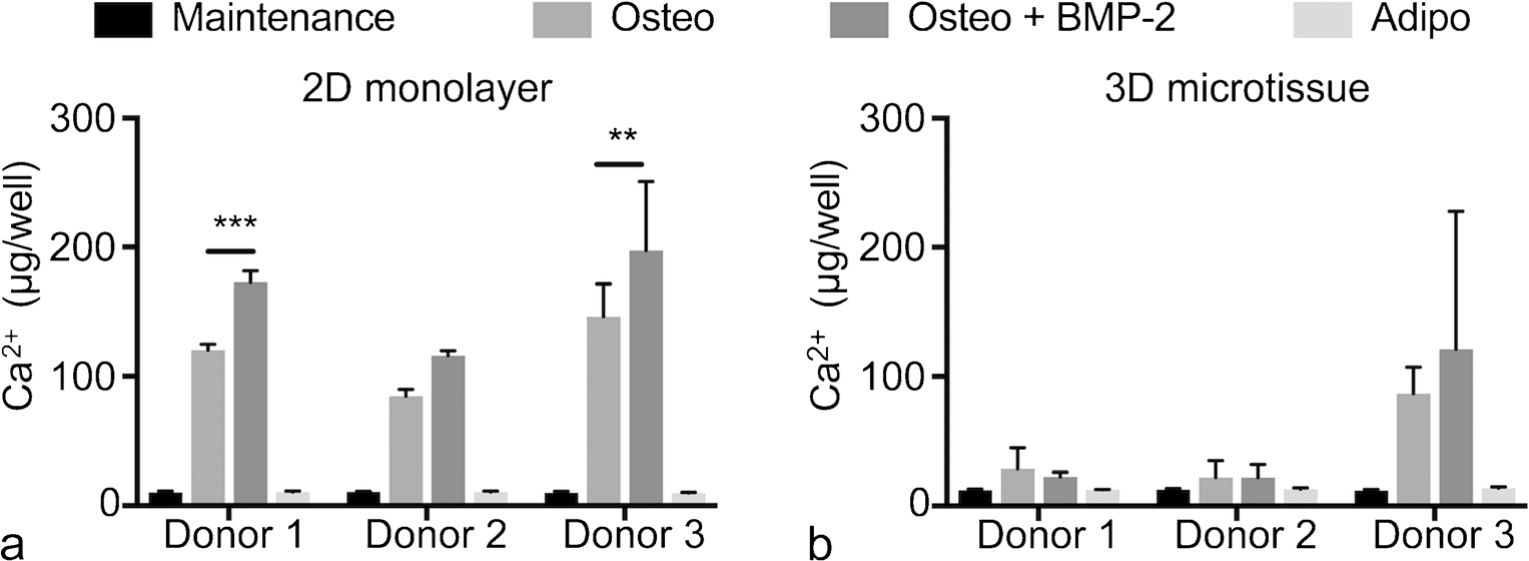
Calcium content in 2D monolayer (a) and 3D microtissue (b) from BMSC donors 1, 2 and 3 after 21-day culture. Data were evaluated using two-way analysis of variance (ANOVA) in Prism software (version 7.0, GraphPad). (** *P* = 0.0011 and *** *P* = 0.0007)

Finally, we quantified the calcium content in 2D monolayer and 3D microtissue cultures at day 21 (Fig. 7). In 2D monolayers, calcium content was clearly greater in cultures maintained in osteogenic medium, plus or minus BMP-2, relative to either maintenance or adipogenic medium. The addition of BMP-2 to the osteogenic induction medium increased calcium content, relative to no BMP-2, which was statistically significant in BMSC cultures derived from donors 1 and 3. In 3D microtissue cultures, increased calcium content was not observed for BMSC cultures derived from donors 1 and 2 cultured in osteogenic medium, plus or minus BMP-2, relative to control cultures. By contrast, calcium content increased considerably in BMSC cultures derived from donor 3 when cultured in osteogenic medium, plus or minus BMP-2. This increase in calcium content is consistent with the visible matrix accumulation adjacent to microtissues observed for BMSC cultures derived from donor 3 in Fig. 4. BMSC cultures derived from donor 3 also displayed the most consistently intense Alizarin Red S staining across all wells in 2D monolayers (Fig. 5).

### Adipogenic BMSC differentiation in 2D and 3D cultures

**Fig. 8:**
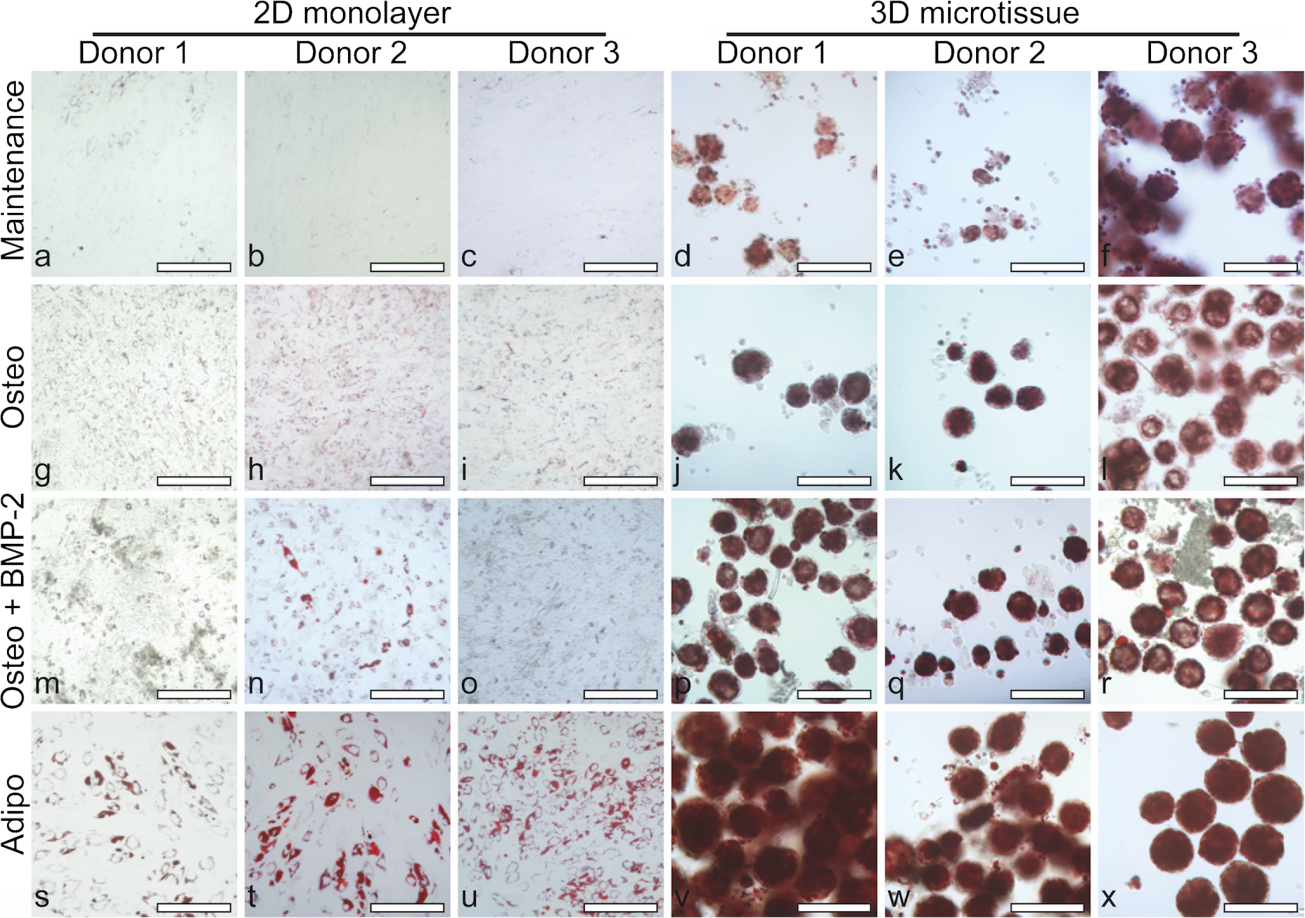
Bright field microscopy to visualise lipid vacuoles (red) by Oil Red O staining in 2D cultures for BMSC donor 1 (a, g, m and s), donor 2 (b, h, n and t) and donor 3 (c, i, o and u) and in 3D cultures for BMSC donor 1 (d, j, p and v), donor 2 (e, k, q and w) and donor 3 (f, l, r and x). Day 21 cultures are shown following growth in maintenance medium (DMEM + 10% FBS) (a–f), osteogenic medium (Osteo) (g–l), osteogenic medium + BMP-2 (Osteo + BMP-2) (m–r) or adipogenic medium (Adipo) (s–x). Scale bars = 200 μm

While BMP-2 is commonly used to drive osteogenic differentiation, reports suggest that BMP-2 can also increase adipogenesis (Gimble et al. 1995; Sottile and Seuwen 2000; Lee et al. 2014). For this reason, we characterised the formation of lipid vacuoles using Oil Red O (Kraus et al. 2016) in 2D monolayer and 3D microtissue cultures. In 2D monolayer cultures, large lipid vacuoles stained with Oil Red O were observed in BMSC cultures derived from all three donors in adipogenic medium, with little or no staining observed in maintenance medium control cultures (Fig. 8). Under high magnification of cells cultured in maintenance medium, very small and faintly stained lipid vacuoles were observed. Such small and faintly stained lipid vacuoles were also observed in osteogenic medium, plus or minus BMP-2, to a greater extent than in maintenance cultures but not nearly as much as observed in adipogenic cultures.

**Fig. 9:**
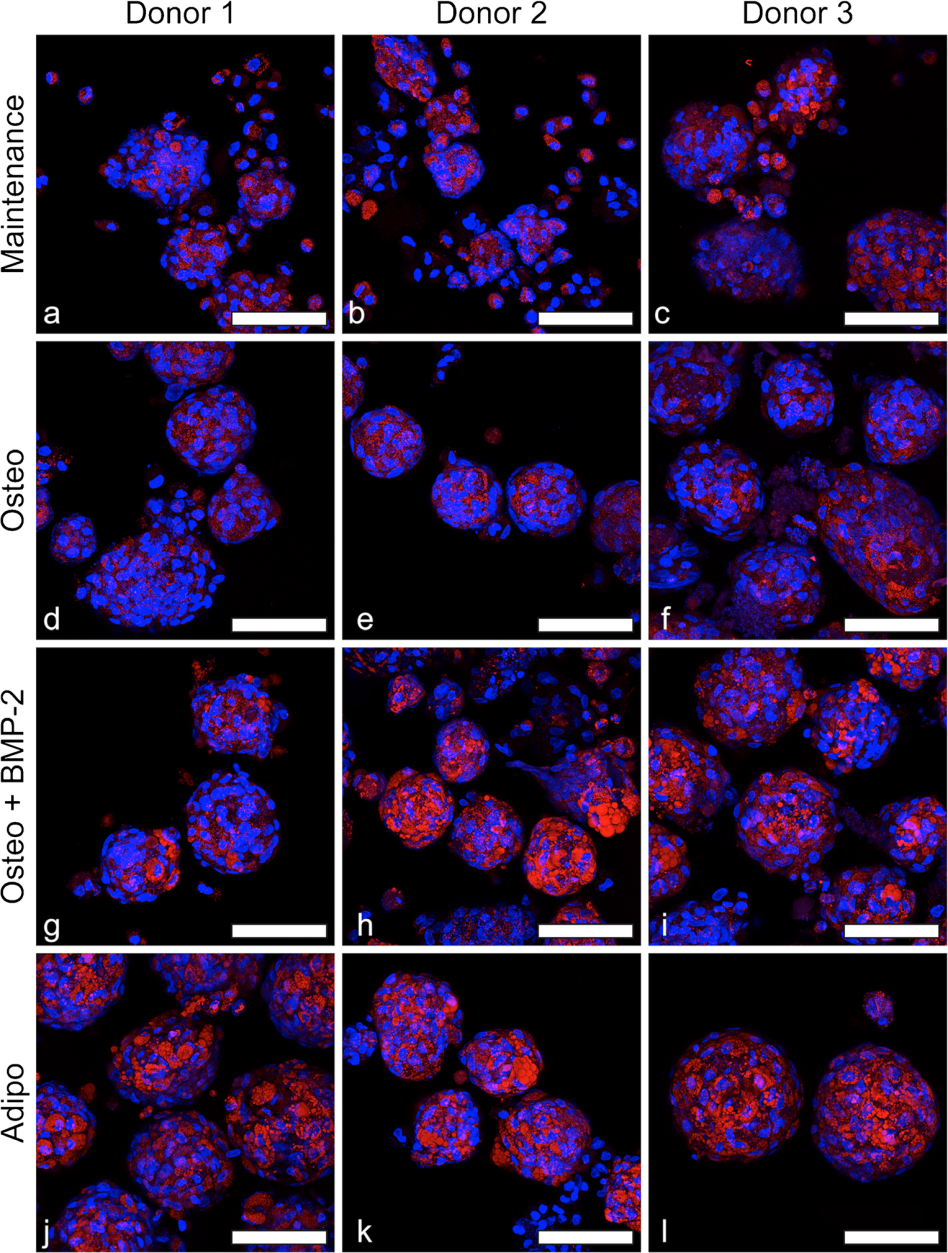
Confocal fluorescence microscopy of lipid vacuoles (red) and nuclei (blue) in 3D microtissues. BMSC donor 1 (a, d, g and j), donor 2 (b, e, h and k) and donor 3 (c, f, i and l) microtissues cultured for 21 days in maintenance medium (DMEM + 10% FBS) (a–c), osteogenic medium (Osteo) (d–f), osteogenic medium + BMP-2 (Osteo + BMP-2) (g–i) or adipogenic medium (Adipo) (j–l). Lipid vacuoles were apparent in BMSC microtissues cultured in all conditions. The prevalence of lipid vacuoles increased in osteogenic medium + BMP-2 relative to maintenance medium. Scale bars = 100 μm

As in 2D cultures, the largest vacuoles and the most intense Oil Red O staining were observed in BMSC microtissues cultured in adipogenic medium (Fig. 8). Additionally, confocal microscopy that was utilised to better visualise the lipid vacuoles in 3D microtissues revealed some lipid staining in all culture conditions (Fig. 9). Small lipid vacuoles were present in all culture conditions, while larger and more intensely stained lipid vacuoles were observed uniformly in adipogenic cultures (Fig. 9j–l). Large lipid vacuoles were also observed in osteogenic induced microtissues, particularly with BMP-2 supplementation (Fig. 9g–i); however, the vacuoles were more heterogenous in size and distribution in osteogenic microtissues, compared to adipogenic microtissues. To our knowledge, the abundance of lipid vacuoles in osteogenically induced BMSC microtissues has not been reported. However, as common histology methods employ tissue processing steps (e.g., freezing or paraffin embedding) that may result in the loss of lipids, the prevalence of lipid vacuoles may be underappreciated in 3D osteogenic tissue cultures.

## Discussion

While BMSC are popular target cells for study in a range of bone repair applications, an optimal mode of delivery has not yet been established. The biological potency of BMSC in the bone repair context might be enhanced by pre-assembling these cells into multicellular bone-like microtissues (Mendez-Ferrer et al. 2010; Pinho et al. 2013), for use as building blocks in bone defect repair. Because of their small diameter, BMSC microtissues may be compatible with minimally invasive orthoscopic delivery into small volume defects such as non-union bone fractures (Calori et al. 2011). The recent surge in the development of 3D culture platforms could make microtissues accessible for therapeutic applications in the near future. To test the feasibility of manufacture and the suitability for bone repair applications, we manufactured hundreds of BMSC microtissues using a high-throughput microwell platform and characterised their osteogenic induction outcomes.

In optimisation studies, we characterised BMSC microtissue diameter and the relative size of individual cells in the microtissues over extended culture periods. Similar to previous reports (Bartosh et al. 2010; Baraniak and McDevitt 2012), we observed the compaction of individual microtissues over the culture period. Eychmans and Chen recently posited that “…cells can spontaneously undergo compaction, a process wherein cells adhere to one another and/or to their surrounding ECM, and contract, thereby increasing the density of the microtissue” (Eyckmans and Chen 2017). We additionally observed a concomitant decline in DNA content over the culture period and a reduction in the volume of the individual cells within the microtissues. A reduction in BMSC size in microtissues has been previously associated with the exit from the proliferative cell cycle of cells within microtissues (Bartosh et al. 2010). We observed that BMSC proliferation was restricted to the periphery of microtissues at early time points, which had largely ceased by day 14. Restricted cell proliferation to the periphery of microtissues is a commonly reported pattern (Sutherland 1988; Chambers et al. 2014).

We evaluated four culture conditions in 2D monolayers or in 3D microtissues, using three unique BMSC donors. As we were specifically interested in evaluating BMSC osteogenesis in 3D microtissues, our media conditions included (1) maintenance medium, (2) osteogenic medium, (3) osteogenic medium supplemented with BMP-2 and (4) adipogenic medium. In both 2D monolayers and 3D microtissues, no bone-like mineralised matrix was observed in maintenance medium controls or adipogenic induced cultures, as expected. In response to osteogenic or osteogenic + BMP-2 medium, both 2D monolayer and 3D microtissue cultures formed an appreciable amount of mineralised matrix. In 2D monolayers, mineral matrix deposition occurred as a layer of crystalline nodules, which over time, formed a continuous mineralised matrix layer. In 3D microtissues, mineralised matrix was deposited as nodules within microtissues, as well as nodules that appeared separated from the bulk microtissue mass. This observation has not been previously reported, to our knowledge. Confocal microscopy analysis of hydroxyapatite-stained microtissues and adjacent nodules that were physically separated from the bulk microtissue mass revealed that much of the non-integrated mineral-rich matrix was formed around what appeared as heavily fragmented nuclei. This suggests that mineralised matrix accumulation during osteogenesis may be associated with cell death, consistent with previous in vitro studies (Fujita et al. 2014). This non-integrated matrix could be viewed as an inefficiency of the microtissue model to retain all matrix produced in a continuous tissue mass. Because BMSC do not divide markedly in 3D microtissue cultures, possibly due to the lack of cell tension normally provided by stiff 2D tissue culture plastic (Eyckmans and Chen 2017), replenishing or maintaining a viable cell pool may also be compromised in this system.

The addition of BMP-2 to osteogenic induction cultures improved bone-like matrix accumulation in 2D monolayer cultures, which was confirmed by calcium quantification (statistically significant for two of three BMSC donors). In 3D microtissues, the addition of BMP-2 to osteogenic cultures resulted in variable outcomes amongst the different BMSC donors and calcium quantification showed no statistical difference. However, because of the lack of mineralised matrix incorporation into the bulk microtissue masses, it is possible that much of the mineralised matrix may be lost over culture time as a result of media exchanges. In a therapeutic setting, it may be more useful to perform short induction periods and allow for much of the mineralisation to occur at the site of bone injury, where the matrix might be integrated and retained in the local bone tissue more effectively.

Here, we evaluated osteogenesis in 3D microtissues by culturing BMSC in osteogenic medium with and without BMP-2 supplementation and compared these to traditional 2D monolayer cultures. BMP-2 has been most widely attributed to enhancing osteogenic differentiation and is used clinically at supra-physiological doses to facilitate bone repair (Hamilton et al. 2008; Ong et al. 2010). However, it has also been reported that BMP-2 can enhance adipogenesis (Sottile and Seuwen 2000; Zehentner et al. 2000). We found that induction with osteogenic medium, with or without BMP-2, does induce bone-like matrix formation but it also increased adipogenesis, more profoundly in 3D microtissue cultures than in 2D monolayers. Lipid vacuoles were identified in 3D microtissues in non-induced and osteogenic-induced cultures. Clear visualisation of lipid vacuoles in 3D tissues required the use of confocal microscopy. It is possible that the presence of lipid vacuoles in osteogenically induced tissues is under reported when the tissues are only characterised following histological sectioning, where lipid content is likely to be lost or less appreciable than whole tissue staining followed by confocal analysis. We recommend that researchers consider this possibility, and where possible assess whole tissues or intact pieces with Oil Red O staining followed by confocal fluorescence microscopy.

BMP-2 supplemented osteogenic-induced cultures showed a higher frequency of large lipid vacuoles, compared to cultures in maintenance medium and osteogenic medium without BMP-2, comparable in size to lipid vacuoles seen in adipogenic-induced microtissues. Seminal work by Engler et al. demonstrated that the culture of BMSC on stiff 2D matrices caused high cytoskeletal stress and promoted osteogenesis (Engler et al. 2006). Studies of BMSC cultured in 3D hydrogels show that adipogenesis is promoted by low modulus gels (Chaudhuri et al. 2016). When cells are cultured in 3D microtissues, cytoskeletal tension is reduced (Eyckmans and Chen 2017) and this may limit the biological capacity of BMSC to generate or retain significant amounts of bone-like matrix in microtissue culture.

A limitation of our study was the use of only 3 bone marrow donors. Our own data indicate that variation between BMSC donors is significant. Donor age or sex might have been identified as influential variables if the study had been replicated with a greater number of donors. However, predicting what these patterns might look like and how many donors would be required to identify significant differences, is challenging. In our study, the BMSC population that deposited the greatest quantity of calcium matrix was derived from a young (21-year-old) female donor. A previous analysis of BMSC from 29 donors found that BMSC populations that divided more rapidly had greater bone-forming capacity and suggested that proliferation rate could be a surrogate of potency (Janicki et al. 2011). They did not observe a gender bias but did observe a negative correlation with donor age. A second analysis of 53 donors found that BMSC from younger females were more often smaller in diameter and divided more rapidly but that the rapid proliferation of female BMSC did not correlate with enhanced osteogenic potential (Siegel et al. 2013). A third analysis of 19 BMSC donors found no correlation between osteogenic potential and age or gender (Siddappa et al. 2007). While all of the donor BMSC populations used in their studies demonstrated osteogenic potential (Siddappa et al. 2007), there was substantial variation in osteogenic gene expression in response to induction factors (specifically dexamethasone). These three studies highlight the significant variability in BMSC osteogenesis and the challenge of attributing variability to a specific donor characteristic (Siddappa et al. 2007; Janicki et al. 2011; Siegel et al. 2013). While our study only utilised 3 BMSC donors, we focused analysis on the critical patterns of cell behaviour that were consistent and most relevant to the BMP-2 response characterisation performed in described microtissue model.

In conclusion, we report that (1) BMSC in 3D microtissue culture resulted in tissue compaction, rather than growth, (2) that not all mineralised bone-like matrix was incorporated in the bulk microtissue mass but could be seen in the vicinity of microtissues as dense particles, (3) that a significant amount of mineralised matrix formed around fragmented nuclei, or dead cells and (4) that a significant amount of lipid vacuoles were observed in 3D microtissues, with the frequency of large lipid vacuoles being more prevalent in BMP-2 supplemented osteogenic cultures. Our original objective was to generate bone-like microtissues from BMSC for use in bone repair. However, our observations suggest that while the idea is conceptually appealing, standard BMP-2 and osteogenic induction medium formulations may not yield a microtissue product that is optimal for bone defect repair. The observations reported in this study may guide the development of research strategies for alternative BMSC microtissue differentiation processes that promote osteogenesis and limit adipogenesis.

## Electronic supplementary material

ESM 1 (DOCX 3588 kb)
