# Supplementary material for: Bone marrow-derived stem/stromal cells (BMSC) 3D microtissues cultured in BMP-2 supplemented osteogenic induction medium are prone to adipogenesis
Source: Cell Tissue Res. 2018 Aug 22;374(3):541–53. doi: 10.1007/s00441-018-2894-y (PMC6267724; doi:10.1007/s00441-018-2894-y)
Supplement: Supplementary file 1 — (DOCX 3588 kb) [file 441_2018_2894_MOESM1_ESM.docx]

**Supplementary Material**

**
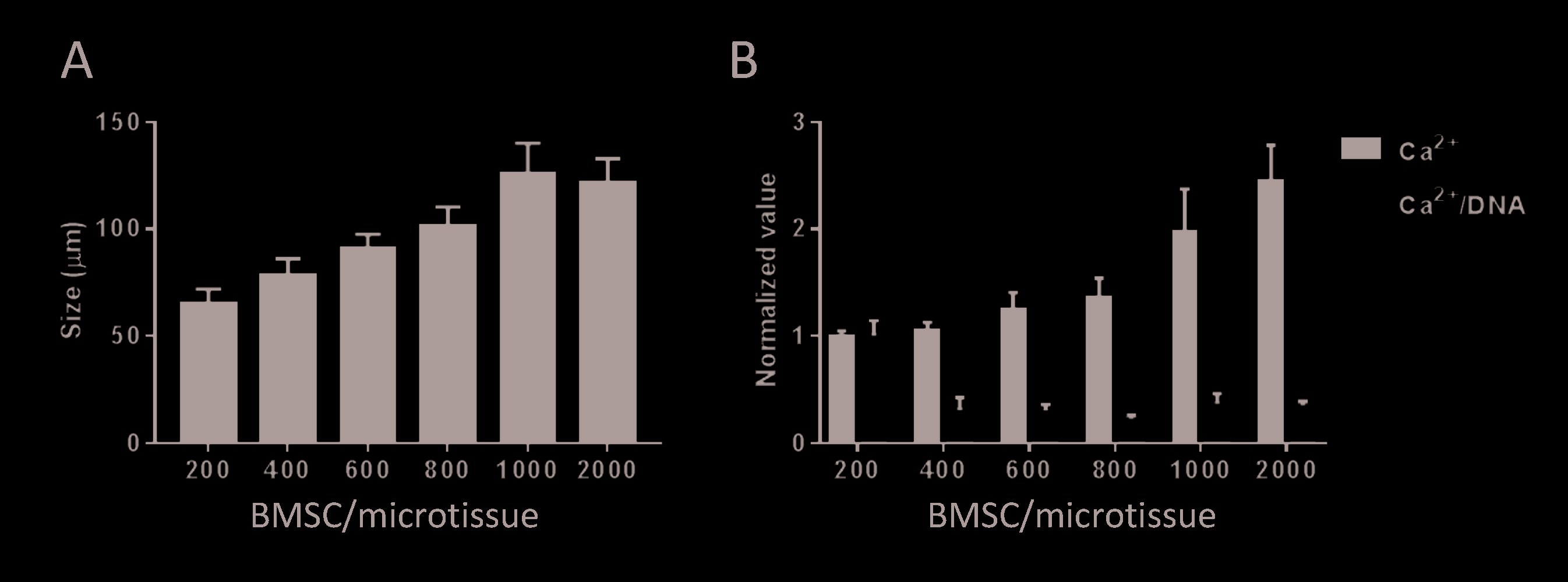
**

**Supplementary Figure 1.** Initial characterisation of BMSC microtissue osteogenesis. **(A)** The diameters of osteogenic induced microtissues formed from 200, 400, 600, 800, 1,000 and 2,000 cells were measured. Varying the initial cell number per microtissue allowed for control of the size of microtissues. Bars represent the mean diameter of 50 microtissues. **(B)** Ca^2+^ and Ca^2+^/DNA ratio was quantified in bone-like tissues and normalized to the spheroids formed from 200 cells/spheroid. Osteogenic induction in 3D spheroids was associated with greater calcium accumulation with increasing cell number per microtissue. However, cells in smaller microtissues accumulated greater calcium per unit of DNA (Ca^2+^/DNA). Similar results were obtained in replicate studies using two different BMSC donors. Each bar is the average of 4 biological replicates and error bars represent standard deviation.

**
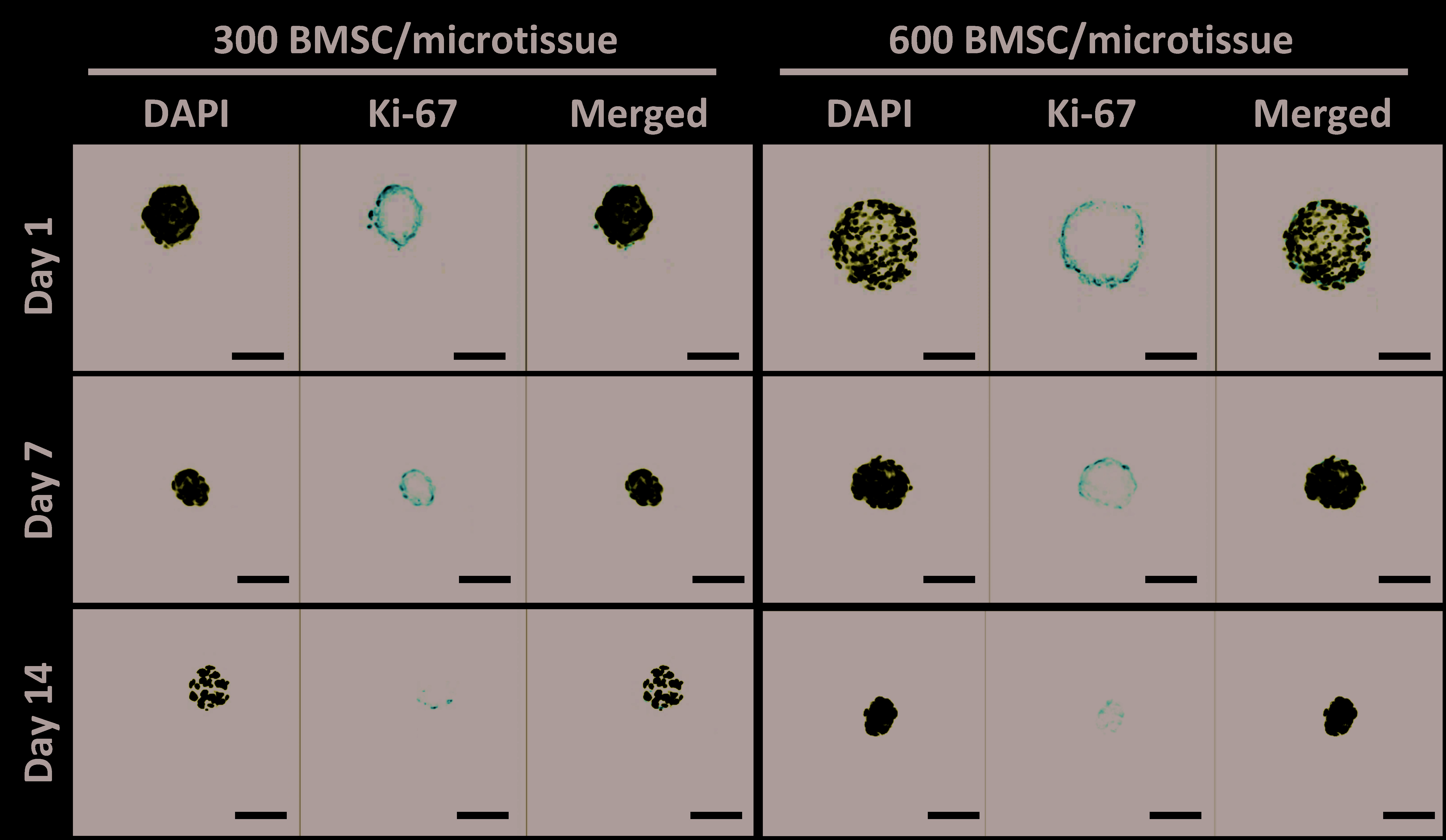
**

**Supplementary Figure 2.** Confocal sections of microtissues (300 and 600 BMSC/microtissues) stained with Ki67 (red) and nuclear stain (DAPI; blue) were acquired on day 1, 7 and 14 of culture. Ki67 (red) staining is localised to the periphery of microtissues, and fades over the culture period. Scale bar = 100 µm.
